# Maladaptive Use of Autobiographical Memory by Bereaved Individuals Across Adulthood

**DOI:** 10.1093/geroni/igaa057.2047

**Published:** 2020-12-16

**Authors:** Tabea Wolf, Veronika Strack, Susan Bluck

**Affiliations:** 1 Ulm University, Ulm, Baden-Wurttemberg, Germany; 2 Ulm University Hospital, Ulm, Baden-Wurttemberg, Germany; 3 University of Florida, Gainesville, Florida, United States

## Abstract

Remembering one’s personal past can serve adaptive psychosocial functions (Bluck, Alea, & Demiray, 2010). Autobiographical remembering has been related to well-being in older age but little research has focused on grief. We address this issue in two studies grounded in the model of reminiscence and health in older adulthood (Cappeliez & O’Rourke, 2006). Participants (aged 18 - 91) completed the Reminiscence Functions Scale and the Inventory of Complicated Grief. Regression analyses show that negative self-related use of memories, but not positive use, is associated with experiencing more grief. Sharing memories with others (pro-social function) is indirectly linked to grief, as mediated by negative self-related uses. These patterns held for autobiographical recall in general (Study 1; N = 51) and when specifically remembering the deceased person (Study 2; N = 49). How adaptively individuals remember their personal past appears linked to the experience of grief, sometimes even years after the loss.

